# New Aspects of Kidney Fibrosis–From Mechanisms of Injury to Modulation of Disease

**DOI:** 10.3389/fmed.2021.814497

**Published:** 2022-01-12

**Authors:** Marcus J. Moeller, Rafael Kramann, Twan Lammers, Bernd Hoppe, Eicke Latz, Isis Ludwig-Portugall, Peter Boor, Jürgen Floege, Christian Kurts, Ralf Weiskirchen, Tammo Ostendorf

**Affiliations:** ^1^Division of Nephrology and Clinical Immunology, RWTH Aachen University Hospital, Aachen, Germany; ^2^Heisenberg Chair for Preventive and Translational Nephrology, Aachen, Germany; ^3^Institute of Experimental Medicine and Systems Biology, RWTH Aachen University Hospital, Aachen, Germany; ^4^Department of Internal Medicine, Nephrology and Transplantation, Erasmus Medical Center, Rotterdam, Netherlands; ^5^Department of Nanomedicine and Theranostics, Faculty of Medicine, Institute for Experimental Molecular Imaging, RWTH Aachen University, Aachen, Germany; ^6^Division of Pediatric Nephrology and Kidney Transplantation, University Hospital of Bonn, Bonn, Germany; ^7^German Hyperoxaluria Center, Pediatric Kidney Care Center, Bonn, Germany; ^8^Institute of Innate Immunity, University Hospital of Bonn, Bonn, Germany; ^9^Institute for Molecular Medicine and Experimental Immunology, University Hospital of Bonn, Bonn, Germany; ^10^Institute of Pathology, RWTH Aachen University Hospital, Aachen, Germany; ^11^Department of Microbiology and Immunology, Doherty Institute for Infection and Immunity, University of Melbourne, Melbourne, VIC, Australia; ^12^Institute of Molecular Pathobiochemistry, Experimental Gene Therapy and Clinical Chemistry (IFMPEGKC), University Hospital RWTH Aachen, Aachen, Germany

**Keywords:** renal fibrosis, myofibroblast, parietal epithelial cell, inflammasome, crystals, lupus erythematodes, deep learning, fibrosis imaging

## Abstract

Organ fibrogenesis is characterized by a common pathophysiological final pathway independent of the underlying progressive disease of the respective organ. This makes it particularly suitable as a therapeutic target. The Transregional Collaborative Research Center “Organ Fibrosis: From Mechanisms of Injury to Modulation of Disease” (referred to as SFB/TRR57) was hosted from 2009 to 2021 by the Medical Faculties of RWTH Aachen University and the University of Bonn. This consortium had the ultimate goal of discovering new common but also different fibrosis pathways in the liver and kidneys. It finally successfully identified new mechanisms and established novel therapeutic approaches to interfere with hepatic and renal fibrosis. This review covers the consortium's key kidney-related findings, where three overarching questions were addressed: (i) What are new relevant mechanisms and signaling pathways triggering renal fibrosis? (ii) What are new immunological mechanisms, cells and molecules that contribute to renal fibrosis?, and finally (iii) How can renal fibrosis be modulated?

## Introduction

Epidemiological studies show that chronic kidney disease (CKD) affects more than 10% of the world's population (1). With renal disease progression, the final common pathway is kidney fibrosis (2). In general, organ fibrosis and the resulting organ failure are estimated to be responsible for at least a third of all disease-related deaths worldwide (3). In addition to a major social burden, organ fibrosis also represents an immense economic burden for all nations' healthcare systems. It has proven to be the common endpoint and result of excessive wound healing. In the kidney, this results mainly in glomerulosclerosis, tubular atrophy and dilation, tubulointerstitial fibrosis and capillary rarefaction (2). Fibrosis development usually takes a similar course independent of the underlying organ disease and is therefore particularly suitable as a therapeutic target. However, despite all efforts, there is still an urgent need for better fibrosis diagnostic methods, a better understanding of the molecular mechanisms and consequently new and targeted therapies.

The Collaborative Research Center SFB/TRR57 “Organ Fibrosis: From Mechanisms of Injury to Modulation of Disease” was funded by the DFG for 13 years from 2009 to 2021. The aim was to face the challenge of liver and kidney fibrosis. The overarching subject areas of the SFB/TRR57 were (i) initiation of fibrosis, (ii) immunological mechanisms and (iii) repair and modulation of fibrosis. This overview specifically summarizes key research results of the consortium concerning kidney fibrosis. We are aware that there are many further important pathways leading to kidney fibrosis that have now been confirmed and established in many studies worldwide. These include, for example, the signaling pathways of numerous growth factors and cytokines, complex pathways controlled by long non-coding RNA or metalloproteinases and many others that we cannot deal with in this review and for which we would like to refer to excellent recent reviews (2, 4–8).

## Initiation of Kidney Fibrosis

**Figure 1:**
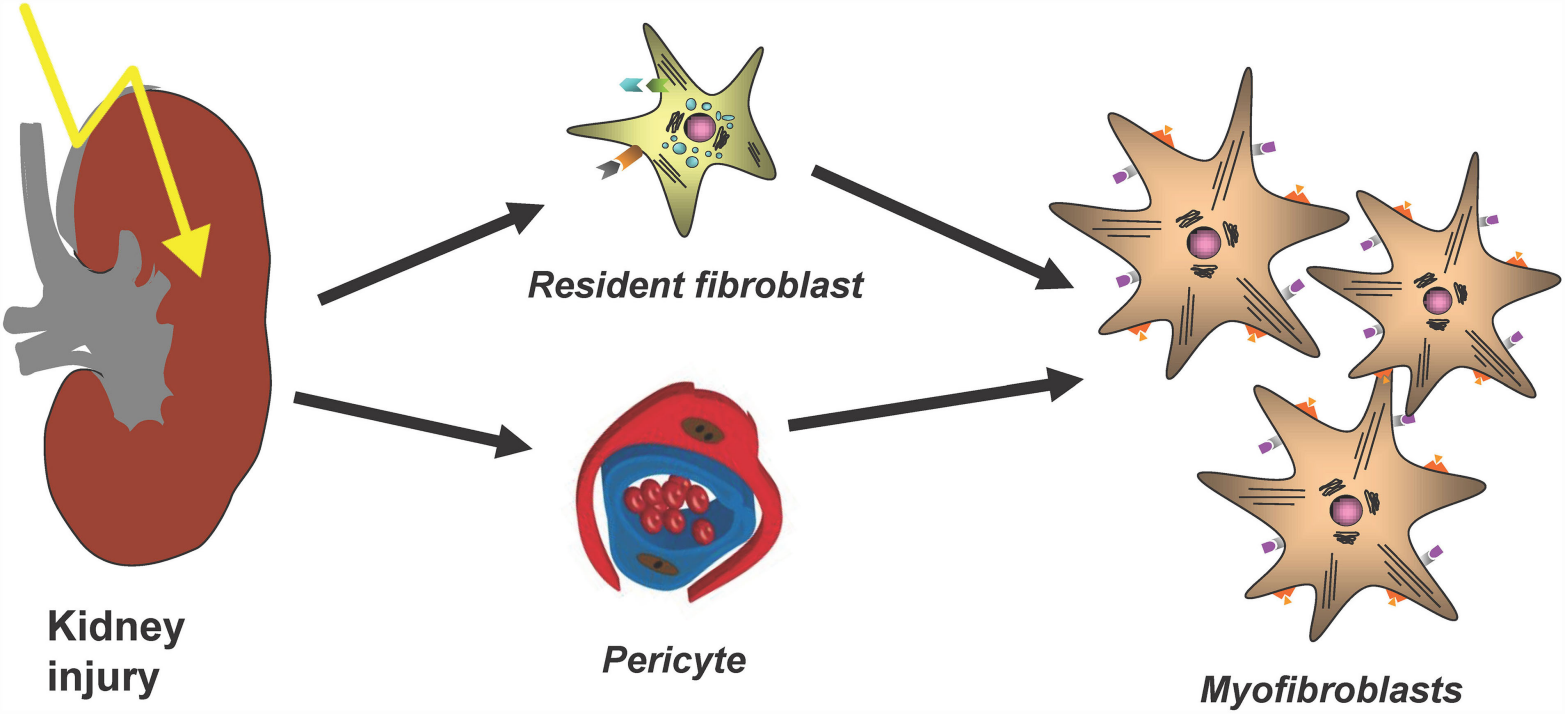
The main cellular source of kidney myofibroblasts following kidney injury and fibrosis progression are resident renal fibroblasts and pericytes.

The major cellular player in the development and progression of kidney fibrosis is the myofibroblast (Figure 1). However, the cellular origin of renal myofibroblasts is still unclear. Up to now different cell types like fibroblasts, pericytes, proximal tubular epithelial cells, bone marrow-derived mesenchymal stem cells (MSC), hematopoietic lineage-derived fibrocytes and macrophages were discussed. This chapter will summarize recent experiments that addressed this question with new, high-resolution techniques and that provide new insights into the role of different populations of mainly fibroblasts and pericytes in the initiation of renal fibrosis.

### Dissecting Pathways Initiating a Pro-fibrotic and Anti-angiogenic Injury Response in Perivascular Myofibroblast Precursors in Kidney Fibrosis

Myofibroblasts are key drivers of kidney fibrosis. This has been shown, for example, by targeting of secreted modular calcium-binding protein 2 (SMOC2), an activator from the transition of fibroblasts to myofibroblasts, which led to an amelioration of kidney fibrosis (9). Gli1^+^ perivascular cells were identified as important myofibroblast progenitors across major organs using genetic fate tracing and ablation experiments (10). Importantly targeting these cells ameliorates fibrosis and stabilizes organ function (10, 11). Thus, Gli1^+^ cells are an important myofibroblast progenitor pool, representing a distinct stromal subtype. Importantly, it was also shown that Gli1^+^ cells detach upon injury from peritubular capillaries and that genetic ablation of Gli1^+^ cells in homeostasis induces capillary loss and hypoxic tubular injury with subsequent fibrosis (12).

**Figure 2:**
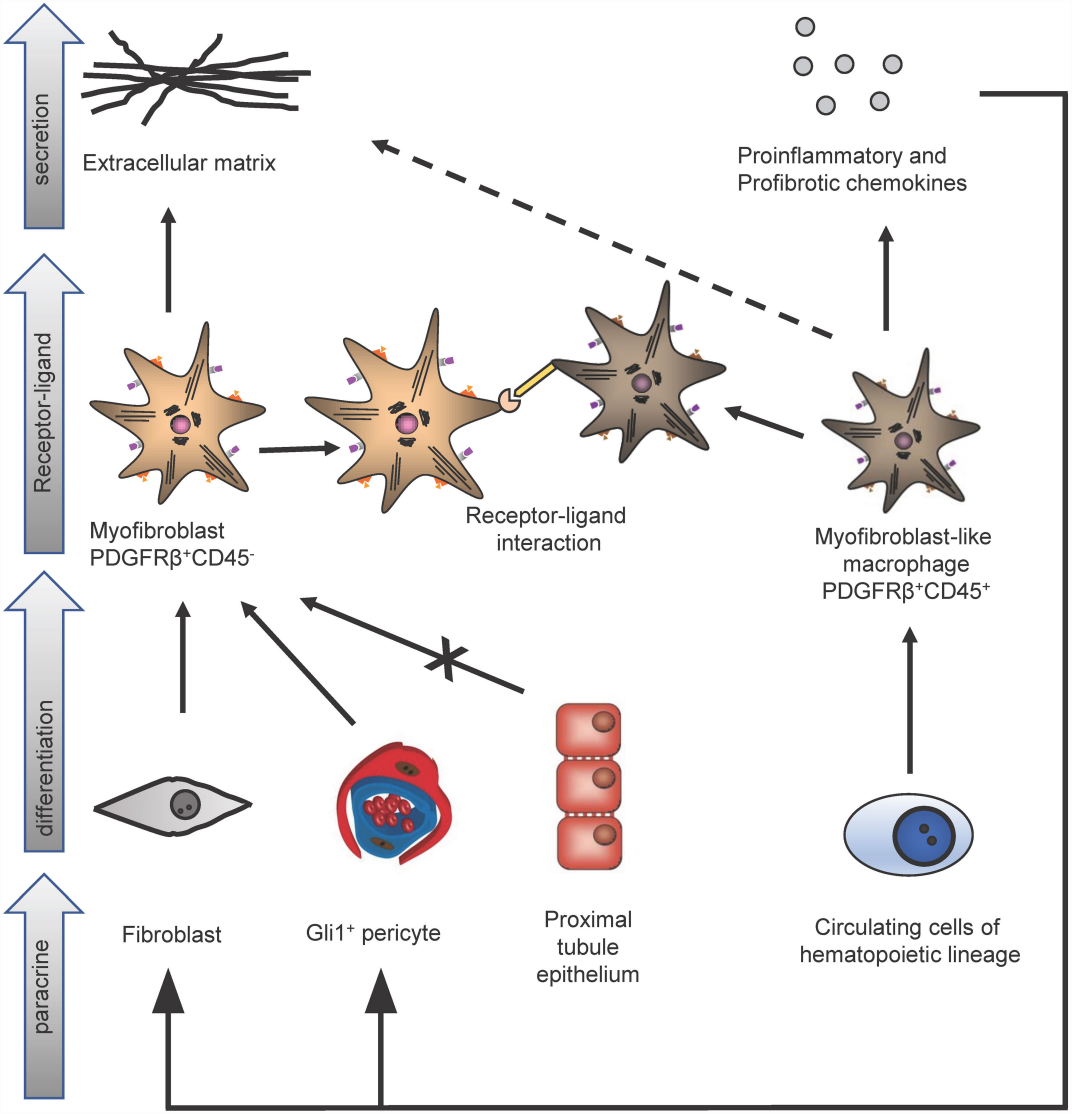
Identification of different kidney myofibroblast subtypes in renal fibrosis. In kidney fibrosis two different types of myofibroblasts could be discriminated: PDGFRβ^+^/CD45^−^ myofibroblasts and PDGFRβ^+^/CD45^+^ myofibroblasts-like cells. PDGFRβ^+^/CD45^−^ fibroblasts originate from pericytes and fibroblasts but not from proximal tubule epithelium and secrete extracellular matrix proteins. Circulating cells of the hematopoietic lineage differentiate in PDGFRβ^+^/CD45^+^ myofibroblasts-like cells. These myofibroblasts-like cells secrete extracellular matrix, pro-inflammatory, profibrotic chemokines and interact *via* receptor-ligand-binding with PDGFRβ^+^/CD45^−^ myofibroblasts.

However, importantly Gli1^+^ cells contribute only to about half of all myofibroblasts in the kidney and thus the origin of the other half remained unclear. The contribution of circulating progenitor cells, e.g., MSC, fibrocytes and macrophages, to the myofibroblast pool was investigated in different studies using bone marrow transplantation and/or confocal microscopy techniques. Bone marrow transplantation experiments were restricted to analyze the role of hematopoietic stem cells in the development of myofibroblasts, since MSC do not engraft well after transplantation (13). Confocal microscopy studies were limited, because hematopoietic stem cells and myofibroblasts were discriminated just by cellular morphology, in particular: the thin branched architecture and their abundance in the renal interstitium after injury (10). A parabiosis model in combination with a generalized genetic fate tracing of all cells and the induction of kidney fibrosis enabled to investigate if any circulating cells contribute to the kidney myofibroblast pool (14). Rosa26CreER;tdTomato-CD45.2^+^ mice were treated with tamoxifen to genetically label all cells and subjected to parabiosis surgery with B6-CD45.1^+^ mice. Kidney fibrosis was induced in the B6-CD45.1^+^ parabiont after 4 weeks by unilateral ureteral obstruction (UUO). Analyzing the fibrotic kidney showed, that a minor fraction of renal myofibroblasts (as defined by α-smooth muscle actin expression) originated from circulating cells of the hematopoietic lineage (14). Characterization of circulation-derived platelet-derived growth factor receptor type β (PDGFRβ)^+^/CD45^+^ myofibroblasts using single cell RNA-sequencing (scRNA-seq) indicated that these cells represent a population of pro-inflammatory monocytes. Importantly, the data showed that these cells do express some extracellular matrix but at far lower levels as compared to resident PDGFRβ^+^CD45^−^ myofibroblasts (Figure 2). A receptor ligand analysis using the scRNA-seq data suggested that the circulating population primarily acts via proinflammatory activation of resident myofibroblasts and does not contribute much to matrix expression (14).

Single nucleus RNA-sequencing technique (snRNA-seq) in fibrotic kidneys after 14 days of UUO in mice in another study identified two different α-smooth-muscle actin-expressing subgroups of myofibroblasts (15). One was a mannose-2 receptor-expressing subgroup that was believed to be able to reduce renal fibrosis by binding and internalizing collagen. In addition, a tenascin-C-expressing subgroup was discovered which, on the other hand, probably promotes renal fibrosis (15).

In a next step it was aimed to move from mouse into human. To map the human kidney in an unbiased fashion on single cell level scRNA-seq analyses were performed from patients with and without CKD (16). This enabled mapping of all matrix-producing cells in human kidneys at high resolution, revealing distinct subpopulations of pericytes and fibroblasts as the major cellular sources of scar forming myofibroblasts during human kidney fibrosis. This approach further enabled the identification of potential novel targets and mechanisms. The targets were validated using human induced pluripotent stem cell (iPSC) derived organoids.

Also slightly increased ECM expression in additional cell-types such as proximal tubule epithelium, endothelium and macrophages were observed (17). The epithelial-to-mesenchymal transition (EMT) of proximal tubular epithelium into myofibroblast has been controversial discussed for more than 20 years. Studies using genetic fate-tracing experiments demonstrated a major contribution (18), no contribution (19) or a minor contribution (20) of EMT. Although a minor matrix production in proximal tubule epithelium could be detected, the proof of a contribution of proximal tubule epithelial cells to the pool of myofibroblasts failed in an inducible genetic fate tracing experiment using bigenic SLC34a1-GFPCreER;tdTomato mice in the murine kidney fibrosis model of UUO (14) as well as in the scRNA-seq study (17). However, injured proximal tubule epithelial cells are still considered important drivers of kidney injury and fibrosis, as e.g., also recently demonstrated by scRNA-seq studies in mouse models of acute kidney injury (21, 22). snRNA-seq in a fibrosis mouse model of chronic aristolochic acid administration identified novel classes of proximal tubular cells associated with kidney fibrosis (23). A scRNA-seq study in kidneys of patients with IgA nephropathy identified a transitional cell type among tubular intercalated cells with fibrosis signatures, suggesting an adverse outcome with interstitial fibrosis (24). All these tubular cells certainly acquire various mesenchymal features but based on the genetic fate tracing studies they do not cross the basement membrane to become interstitial matrix-secreting myofibroblasts.

In summary, the data indicate that renal extracellular matrix-producing myofibroblasts originate from distinct fibroblast and pericyte populations.

## Immunological Mechanisms in Kidney Fibrosis

**Figure 3:**
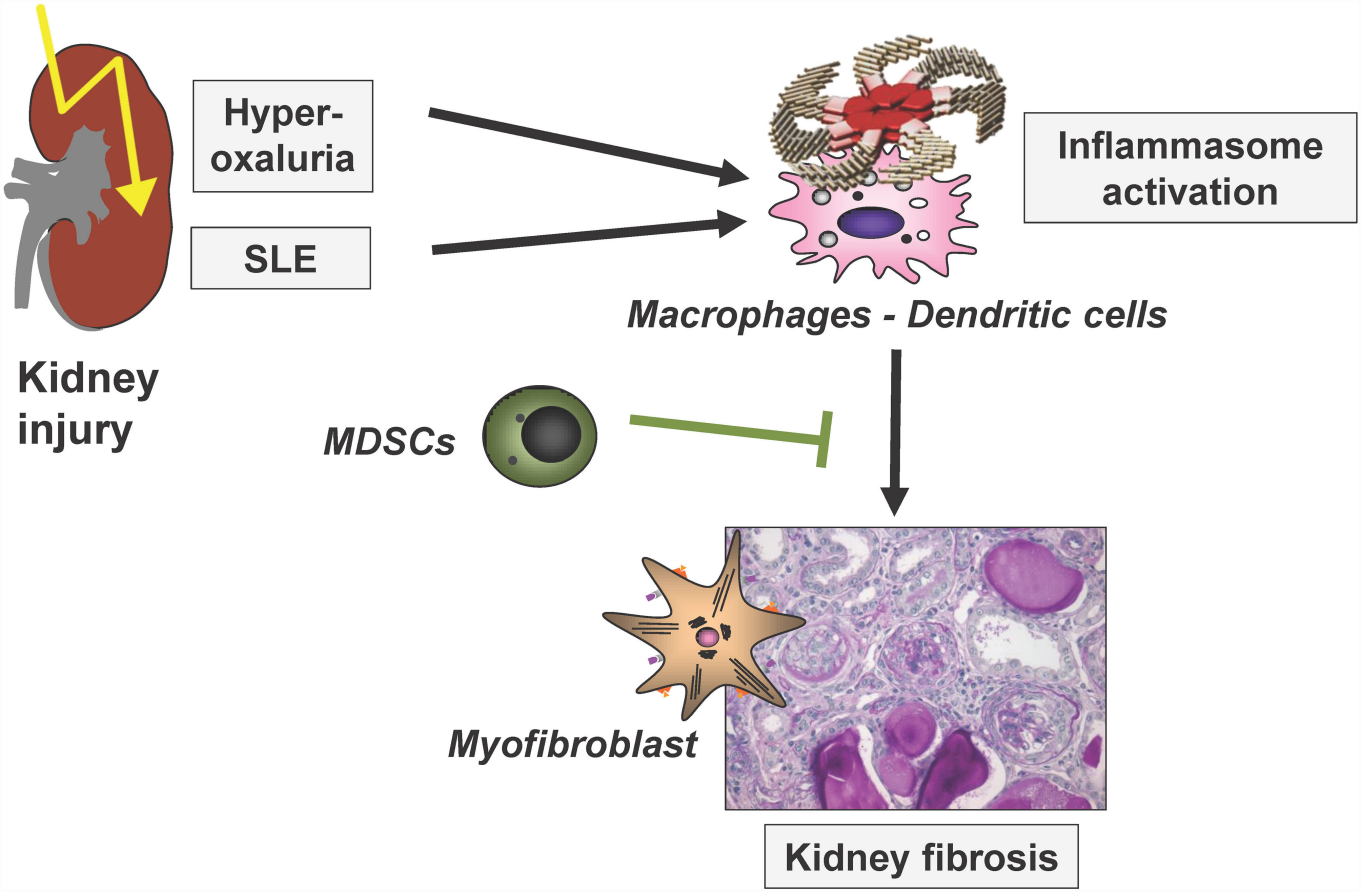
Simplified scheme to illustrate renal inflammasome activation by two disease entities, finally leading to renal fibrosis. SLE, systemic lupus erythematosus; MDSCs, myeloid-derived suppressor cells. Modified based on (25).

Immune-mediated mechanisms are central for the initiation, progression and also limitation of kidney disease and fibrosis development. The following chapter comprises recent studies investigating various central immunological mechanisms in these processes, next to fibrosis finally also leading to kidney dysfunctions at the latest in the terminal phases of most kidney diseases. In addition, studies investigating immunological mechanisms limiting renal fibrosis progression were discussed. These include analyses on the role of a dendritic-cell-specific chemokine receptor in glomerulonephritis and on the mechanisms of inflammasome activation in crystal-induced nephropathy and in lupus nephritis as illustrated in Figure 3.

### Inflammasome Activation and Crystal-Induced Renal Fibrosis in Primary Hyperoxaluria

The kidney harbors an extensive network of mononuclear phagocytes consisting of macrophages and DCs, which regulate innate and adaptive immune responses, respectively, in sterile and infectious kidney inflammation (26). Kidney DCs specifically depend on the chemokine receptor CX3CR1 (27) and mice deficient for this receptor possess far less DCs in the kidney, but not in other organs. Consequently, glomerulonephritis models were attenuated and fibrosis resulting from the ensuing chronic inflammation reduced (27). Based on this, it was proposed that CX3CR1 inhibition might be a suitable therapeutic strategy against glomerulonephritis because of its specificity for renal DCs (27, 28).

Both DCs and macrophages can promote inflammation by activating inflammasomes (29). The central inflammasome component NACHT, LRR, and PYD domains-containing protein 3 (NLRP3) together with the adaptor protein ASC and caspase-1 forms a multimolecular platform that proteolytically activates pro-forms of interleukin (IL)-1β and IL-18 (29). Activation of the NLRP3-inflammasome requires two signals: the first can be induced by various microbial or danger-associated molecular patterns stimulating NLRP3 production, and the second can be triggered by crystalline particles, which causes inflammasome assembly. The kidney is particularly prone to NLRP3 inflammasome activation because of its function to concentrate the glomerular filtrate, which favors crystal precipitation when solubility coefficients are exceeded (30). It was demonstrated that a soluble NLRP3 inflammasome inhibitor (31) attenuated inflammasome activation and the resulting fibrosis in a murine model of crystal nephropathy induced by oxalate- or adenine-rich diets (32). Reduced inflammasome activation was thereby visualized in living mice with a new imaging system that employed a proteolytic luciferase-based inflammasome reporter. Urinary IL-18, but not IL-1, concentrations were elevated suggesting potential use as a biomarker. In summary, this therapy suppressed the development of fibrosis, but not the resolution of established renal fibrosis.

**Figure 4:**
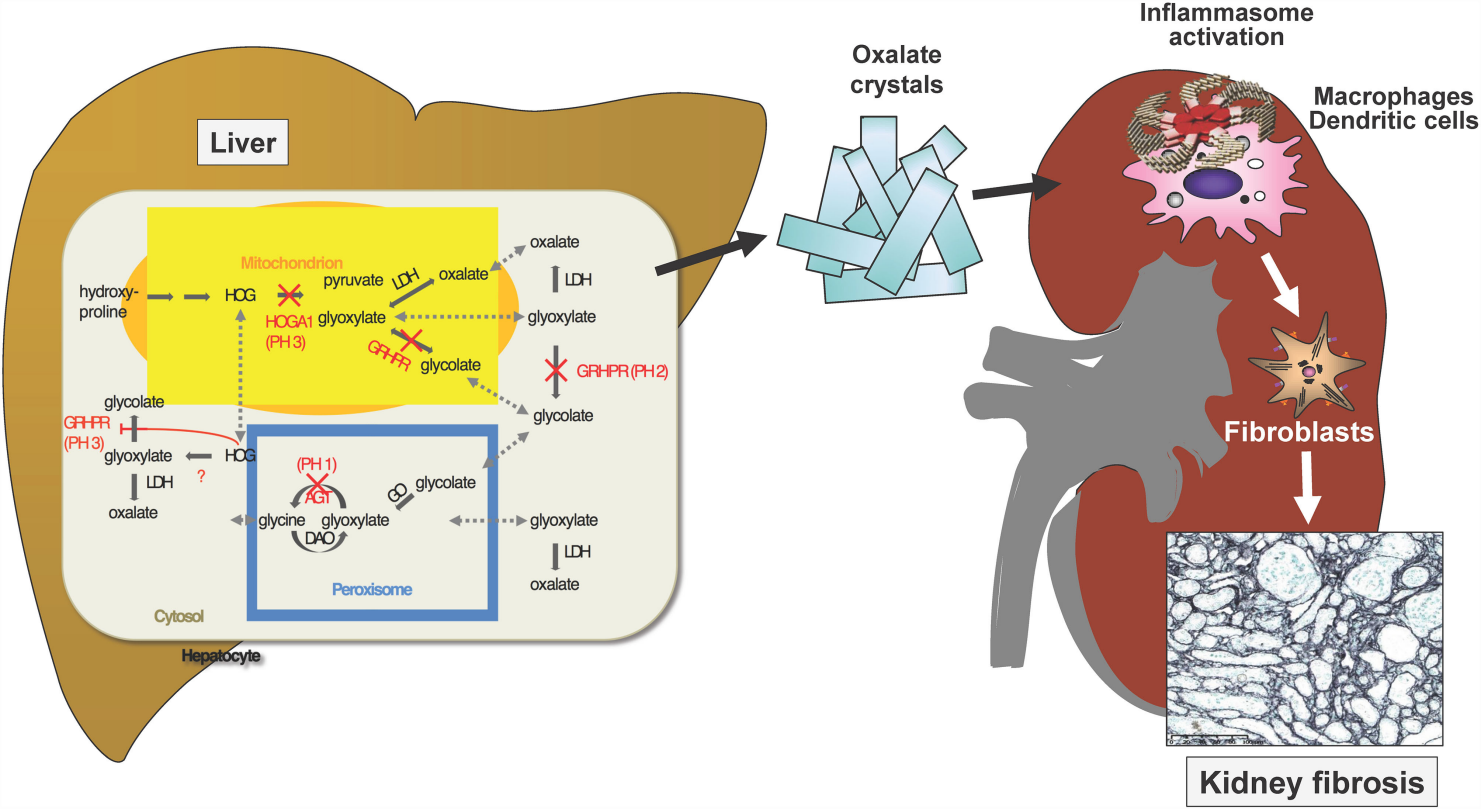
Three known gene defects in hepatocytes cause oxalate accumulation in primary Hyperoxaluria I-III. Ca-Oxalate crystals precipitate in the kidney where water gets reabsorbed and the solubility coefficient of this salt is exceeded. The crystals activate the inflammasome in renal macrophages and dendritic cells, which stimulate interstitial fibroblasts to deposits collagen, resulting in kidney fibrosis.

The primary hyperoxalurias (PH) comprise three inborn errors of the glyoxylate metabolism in the liver (Figure 4). Endogenous oxalate overproduction leads to significant hyperoxaluria, which is culprit of recurrent kidney stones or progressive nephrocalcinosis. This frequently causes even early end-stage renal failure and then also affects other organs, for example heart, bones, retina, muscles and skin by systemic oxalate depositions. The three currently known types of PH are distinguished by different genetic defects, different urinary excretion of other metabolites next to oxalate and of course, by their different clinical courses (33). Type 1 is with 70–80% the most prevalent form and results from a defective or mitochondrial mis-localized, but normally peroxisomal enzyme (AGT; alanine-glyoxylate-aminotransferase). It causes recurrent urolithiasis and/or severe nephrocalcinosis. Therapeutic options were scarce and only included hyperhydration, alkaline citrate medication and vitamin B6, which is a cofactor to AGT and helps preventing the mislocalization of AGT. The only curative option, however, was liver/kidney transplantation as combined or sequential procedure. Type II is a consequence of defective glyoxylate reductase/hydroxypyruvate reductase (GRHPR). It is less prevalent (<10%), but also leads to CKD in about 50% of patients and ESRD in about 25%. In ESRD isolated kidney transplantation is sufficient in most patients, but in some liver/kidney transplantation is necessary due to the severity of the clinical course. Type III results from dysfunctional HOGA1 (4-hydroxy-2-oxoglutarate aldolase type 1) and is the second most frequent type in Germany (17%). It causes sometimes severe recurrent urolithiasis and nephrocalcinosis in about 10% of patients. So far it was regarded to have a less problematic course, but according to new registry data it is known, that also adult patients remain active stone formers and that about 21% of patients develop > stage 2 CKD (17). Also, patients with end stage renal failure are now observed (34).

Mouse models exist that mimic the biochemical and clinical traits of human disease (35). These models are superior to the alimentary models of crystal nephropathy that were used in the past, because the oral application of oxalate or adenine in these models not only elevate circulating levels of these metabolites for triggering crystal formation, it also affects the intestine and its microbiome, resulting in higher levels of circulating microbial compounds, which represents signal one in inflammasome activation, thereby introducing an unphysiological factor into the model. Preliminary studies indeed showed that kidney disease in Agtx1 mice is milder than after oral application of large oxalate amounts, at least in the first months of age. Future studies may use dietary adjustments to accelerate the disease model for mechanistic or therapeutic studies. It is tempting to speculate that inhibition of the inflammatory consequences of crystals through inflammasome activation may synergistically complement existing therapeutic strategies aimed at preventing oxalate accumulation.

Current treatment options were not able to halt disease progression over time in most of the patients. With the exception of vitamin B6 responsive PH 1 patients, all others were just treated as an idiopathic CaOx stone former, who, of course, has a minor risk of recurrence as compared to the PH's. Therefore, new treatment options were clearly needed (36). Such will be able to prevent damage (renal and later systemic oxalosis) and therefore lead way to protection of kidney function and hence a huge amelioration of clinical follow-up. First new treatment available are the RNA interference (RNAi) medications, which became available early 2021. RNAi selectively blocks the endogenous oxalate production in the liver by silencing either glycolate oxidase (GO, upstream pathway, Oxlumo^TM^, for PH 1), or liver specific lactate dehydrogenase A (LDHA, downstream pathway, Nedosiran, theoretically for PH1-3), (37, 38). The oxalate-degrading bacteria (Oxalobacter) included in the first-in-class product Oxabact using oxalate as its sole source of energy were extensively studied in patients with PH. There were positive reports in PH patients on dialysis (39). However, the recent pivotal study did not reach the aimed end-point (press release Oxthera). Future treatment options will definitively also include oral GO blocking agents, for which a phase I study will start soon, but also CRISPR/Cas and gene therapy, in which clinical trials are not yet on the horizon.

### Role of Inflammasome Activation in Lupus Nephritis

Systemic lupus erythematodes (SLE) is a chronic, systemic autoimmune disorder that is characterized by the presence of antinuclear autoantibodies (ANAs), anti-DNA and anti-Sm autoantibodies, and the development of multiple organ pathologies that can be life-threatening. In most SLE patients, the pathogenesis is multifactorial, and SLE is triggered by a combination of genetic predispositions and environmental factors. Insights in disease susceptibility in non-familial SLE forms propose that the main drivers of SLE pathogenesis are the excessive presence of autoantigens derived from dying cells together with a disproportionate recognition of host-derived nucleic acids through toll-like receptors (TLRs) by the innate immune system (40). In line with this hypothesis is the notion that overexpression of membrane-bound Fas ligand drives pathology in experimental SLE (41). This excessive accumulation of autoantigens can be induced by changes in death pathways such as apoptosis, pyroptosis, and NETosis, as well as through defects in the clearance of dying cells (42). These autoantigens can be presented by follicular dendritic cells to autoreactive B cells in germinal centers of secondary lymphoid organs leading to the loss of B cell tolerance and the production of autoantibodies. These autoantibodies form immune complexes (ICs) with cellular autoantigens, consisting of nuclear components or nucleic acids leading to inflammation and organ damage. Phagocytes take up the ICs and respond with pro-inflammatory cytokine secretion, chemokine production, and factors leading to tissue remodeling and organ damage. Also, plasmacytoid dendritic cells produce high amounts of interferon-α upon IC uptake, thereby contributing to an interferon signature observed in some SLE patients (43).

Lupus nephritis (LN) is one of SLE's most severe manifestations and occurs in 40~70% of patients with SLE. LN can lead to advanced sclerosis in the kidneys with a fibrotic transformation of more than 90% of glomeruli. Even with increased knowledge of disease pathogenesis and improved therapies, LN is associated with considerable morbidity and mortality. Within 10 years of an initial SLE diagnosis, 5–20% of patients with LN develop end-stage kidney disease that is life-limiting (43). A better molecular understanding of LN is expected to provide the rationale for novel therapeutic approaches. In the last years, research has helped to define the pathogenetic mechanisms of renal manifestations. Here, a multifaceted role of type I interferons was discovered. New insights have been gained into the contribution of immune complexes containing endogenous RNA and DNA in triggering the production of type I interferons by dendritic cells via activation of endosomal toll-like receptors.

Genetic studies in various SLE populations have identified many predisposition loci within the endosomal TLR signaling pathways, and the same was observed in several genetic models of murine SLE (42). Here, disease onset and progression in pristane-injected mice are highly dependent on endosomal TLRs such as TLR7 together with type I IFNs (44). Pristane-injected *Tlr*7^−/−^ B6 mice show reduced IgG depositions in the kidneys, inducing less severe nephritis and improving survival rates. That demonstrated that *Tlr7*-deficient mice are protected from murine SLE. The results were obtained from several genetic murine SLE models, including MRL/lpr, B6/lpr, Ali5 B6, Nba2.Yaa, B6.Nba2, and WASp-deficient B6 mice. TLR9-deficiency, in contrast, showed drastically increased SLE disease severity (45). This TLR7-TLR9 paradigm might be explained by the more pathogenic RNA containing immune complexes in TLR9 deficient mice that deposit in the kidneys leading to more severe renal disease (46). Of note, small molecule antagonists of TLRs involved in SLE pathogenesis have been developed (47). It is not entirely clear how TLR9 exerts its protective role in SLE pathogenesis. One hypothesis was that myeloid-derived suppressor cells (MDSCs) might be involved in this protection.

MDSC have been described to be important during pathogenic conditions, such as inflammation (48), autoimmunity (49) or cancer (50). This heterogeneous cell population is characterized by the expression of CD11b, Ly6C, and Ly6G. Depending on the expression level of Ly6G and the morphology, MDSC can be further distinguished into Ly6G^low^ monocytic MDSC (M-MDSC) or Ly6G^high^ granulocytic MDSC (G-MDSC). In homeostasis, MDSC is localized in the BM and low numbers in the spleen. MDSC expand and accumulate in secondary lymphatic organs and non-lymphoid tissues, e.g., the kidney during inflammation promoted by cytokines such as IL-1β, IL-4, IL-6, IL-13, and IFN-γ, growth factors (e.g., GM-CSF or VEGF) and TLR signaling (51). Activated MDSCs secrete, for example, arginase-1 and nitric oxide synthase (NOS2) and increase the production of nitric oxide (NO), reactive oxygen species (ROS,) and reactive nitrogen species (RNS) to mediate their immunoregulatory function. Furthermore, MDSC produce the immunosuppressive cytokine IL-10. Together with the ability to induce regulatory T cells, these molecules allow MDSC to regulate innate and adaptive immune cells.

In cancer, MDSCs function is clearly defined as suppressive. Here, they suppress the anti-tumor T cell response. Therefore, cancer therapy uses, e.g., CpG, a TLR9 ligand, to mature the MDSC, thereby eliminate their suppressive capacity leading to an increase in tumor defending CD8 effector T cells.

It remains controversial in SLE and especially LN if MDSC attenuate the disease progression or promote disease progression. Recently, a study identified CD11b^+^Ly6C^high^ monocytes in the pristane-induced lupus model to suppress the T cell response in the early onset of disease progression (52). This was supported by findings where the activation of MDSC with all-trans retinoic acid (ATRA) water led to increased ANA autoantibody production and more severe kidney damage compared to the damage observed in TLR9 deficient mice (data not published). In contrast to these findings, a study using a humanized lupus model, where human MDSC were purified from SLE patients' blood and injected into NOD/SCID mice, showed that monocytic MDSC induced Th17 cell differentiation and thereby promote kidney destruction (53).

This may indicate that in SLE, MDSCs may play a dual role depending on the inflammation or their differentiation stages changed by the conditions of the different microenvironments during disease progression (early vs. late stage) (54). In an early-stage lupus-prone mice study, it was demonstrated that MDSCs were able to inhibit T-cell proliferation. In contrast, at later stages, the suppressive function of MDSCs was lost and replaced by the regulation of Th17 and Treg balance (55). This indicates that various differentiation stages might be responsible for the dual roles of MDSCs. Compared to cancer, the number of MDSCs in SLE was lower, leading to more significant heterogeneity in the myeloid population and variable frequency of MDSCs among myeloid cells explaining the discrepancies.

## Repair and Modulation of Kidney Fibrosis

**Figure 5:**
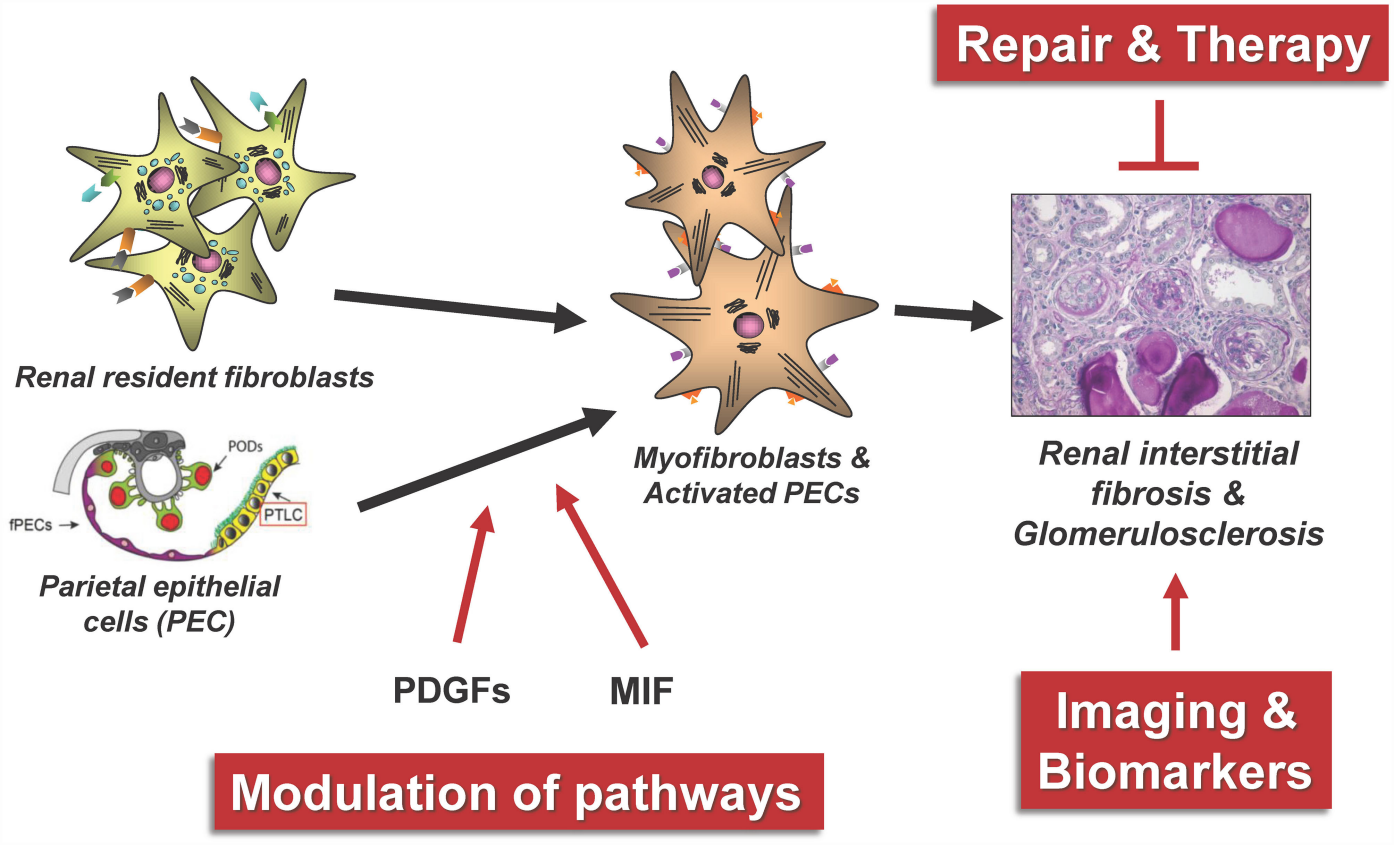
The figure summarizes some central aspects of the repair and modulation of renal fibrosis that have been successfully investigated in our consortium. PODs, podocytes; fPECs, flat parietal cells; PTLC, proximal tubular epithelial-like cell, PDGFs, platelet-derived growth factors; MIF, macrophage migration inhibitory factor.

Next, we will review a selection of novel regulatory mechanisms in kidney fibrogenesis as illustrated in Figure 5. In particular, we will present recent findings on the role of activated parietal epithelial cells (PECs) and the role of growth factors and their receptors from the platelet-derived growth factor (PDGF) family and macrophage migration inhibitory factor (MIF) for the development of renal tubulointerstitial fibrosis and focal segmental glomerularsclerosis (FSGS). The monitoring of possible repair and modulation processes in renal fibrosis furthermore requires the identification of new suitable biomarkers, rapid pathological diagnostic methods and ideally valid non-invasive imaging methods of fibrosis.

### Parietal Epithelial Cells Are Essential for the Formation of Sclerotic Lesions in Focal and Segmental Glomerulosclerosis

FSGS is the most common pathomechanism leading to progressive loss of renal function and finally to end-stage kidney disease. For example, the great majority of glomerulopathies (e.g., IgA nephropathy, membranous glomerulonephritis, but also very common conditions such as hypertensive nephropathy) induce the formation of FSGS lesions, which result in irreversible loss of renal function (60). In all of these conditions, FSGS is termed as *secondary* (i.e., the result of a primary glomerular disease). *Primary* FSGS is a rare condition and describes a form of minimal change disease, which results in formation of FSGS lesion and subsequent loss of renal function (61). The first major step to induce this process is an acute or chronic injury to the epithelial cells (termed podocytes, Pods) on the glomerular capillaries, which are essential for the integrity of the glomerular filter. In the last decade, it has become evident that a second cell type, parietal epithelial cells (PECs), are necessary and required for the formation of FSGS lesions and loss of renal function. PECs also form proliferative lesions (cellular crescents) in rapidly progressive glomerulonephritis (62). Cellular activation of PECs has been demonstrated to be a key pathogenetic event and a prime pharmacological target to develop specific therapies to slow or halt progressive CKD. This chapter summarizes the most relevant recent advances to understand the role of PECs in FSGS.

Podocytes are lost in all glomerulopathies, which may progress to FSGS and end-stage kidney disease. Pods are post-mitotic cells and cannot regenerate such cellular losses. It has been well-documented that beyond a certain threshold of subtotal podocyte loss (about 30%), FSGS lesions form. PECs are a highly attractive candidate to regenerate Pods. PECs line Bowmans capsule, proliferate continuously at a low rate, are in direct continuity with Pods at the vascular pole, where transitional cells can be observed which share characteristics of Pods and PECs (63). Indeed, it could be shown by genetic cell fate tracing that in newborn mice and humans, a significant number of synaptopodin-positive predetermined podocytes reside on Bowmans capsule, which are recruited onto the growing glomerular tuft as the kidneys grow. However, quite early in youth (at the age of about 7 years in humans), this podocyte reservoir is depleted and afterwards no further PECs can be recruited to become podocytes in mice or humans. The groups of Romagnani and Shankland have reported that PEC to Pod transdifferentiation may still occur, e.g., in acute glomerulonephritis (64, 65). Therefore, it remains an open question whether efficient regeneration of Pods might occur from PECs.

**Figure 6:**
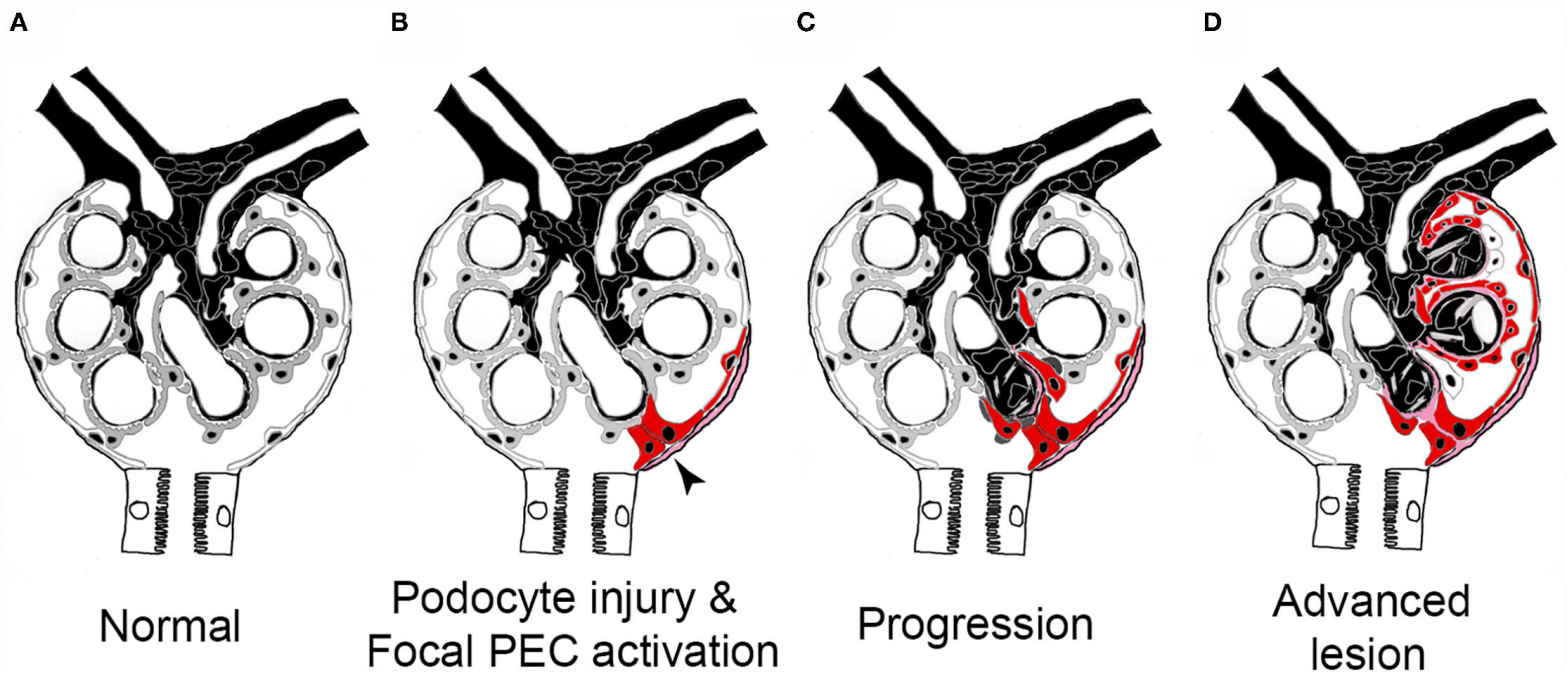
Pathogenesis of sclerotic lesions in FSGS. **(A)** Normal glomerulus. gray, podocytes; white, parietal epithelial cells. **(B)** Podocyte injury or loss triggers focal PEC activation (red), PECs form an adhesion to the capillary tuft (arrow). **(C)** PECs take over parts of the capillary segment, capillaries are lost. **(D)** Advanced stage. Figure taken from (56) with modifications.

In a sequence of studies over the last decade, the pathogenesis of FSGS sclerotic lesion formation could be resolved (Figure 6). To trigger FSGS, an injury or loss of podocytes appears to be required. Next, and compared with the normal physiological state (Figure 6A), PECs become *activated*, which means that their cytoplasm and nucleus enlarge, they express novel protein markers (e.g., CD44, CD9 or pERK), and they migrate and/or proliferate (Figure 6B, red-colored cells). Activated PECs form adhesions to the glomerular tuft (Figure 6B), from where they migrate and expand over the affected segment of the capillary tuft (Figures 6C,D). No FSGS lesions exist without the specific extracellular matrix derived from PECs or without PECs (of note, PECs disappear in some lesions in later stages of the disease). These PECs displace the remaining Pods and deposit their extracellular matrix onto the glomerular basement membrane. As Pods disappear, vascular endothelial growth factor (VEGF) no longer reaches the glomerular capillaries, resulting in capillary loss (66). Ultimately, a segmental scar devoid of Pods or capillaries forms, which is the hallmark lesion in FSGS. Last but not least, the above-described sequence of events resulting in glomerular scar formation also shows similarities to the role of hepatic stellate cells in hepatic fibrosis.

Two studies provide evidence that cellular activation of PECs is necessary and required for FSGS lesion formation. In the first, it was shown that cortisone (glucocorticoids) act directly on PECs and that this results in their deactivation (67). Strikingly, the direct effects of cortisone on PECs appear to be more relevant than its immunosuppressive effects in rapidly-progressive glomerulonephritis. Unexpectedly, genetic or pharmacological inhibition of the glucocorticoid receptor also prevented cellular activation of PECs. In these mice, FSGS lesions (as well as proliferative lesions, i.e., cellular crescents, see below) were ameliorated significantly. In the second study, Tharaux and co-workers observed accidentally, that genetic inactivation of CD9 in the entire mouse or only in PECs prevents cellular activation of PECs in models for FSGS and RPGN (68). Even though the triggering injury (podocyte depletion or deposition of anti-GBM serum) was similar, lesion formation was almost completely prevented in CD9 deficient mice and renal function was preserved. These two studies provide evidence that cellular activation of PECs is necessary and required for the irreversible loss of renal function in secondary FSGS (and also RPGN). Thus, PEC activation is a prime and novel pharmacological target to slow or prevent progressive loss of renal function in the great majority of patients with CKD. One such drug is already known (high-dose glucocorticoids), however this treatment results in unacceptable side-effects when administered over longer periods of time. Specific PEC deactivating agents could be an exciting novel class of drugs in the future.

It is well-known that proximal tubule cells may colonize parts of Bowmans capsule in human patients (termed cuboidal PECs, cPECs). Of note, at the interface to classical flat PECs (fPECs), the first proximal tubule cell shows signs of cellular activation (expression of activation markers, proliferation, etc.). These iPECs are located close to the tubular pole in most cases. It could be now demonstrated that in early primary FSGS, the classical tip lesion emerges from this preactivated iPEC in humans. In summary, this explains the morphology of tip lesions (i.e., adhesion close to the tubular orifice) and why these lesions are the first to form (from pre-activated iPECs) (69).

### Cell-Specific Role of the PDGF System in Renal Fibrosis

PDGF is an important and well-established mediator of organ fibrosis including kidney fibrosis (70). Recent studies identified novel roles and supported translational relevance of PDGF in CKD and renal fibrosis. Inhibition of the PDGF-CC isoform, a ligand of the PDGFRα, using neutralizing antibodies and genetic deletion, significantly ameliorated renal fibrosis and disease progression in Col4a3 deficient mice, a murine model of genetic progressive glomerular disease closely resembling the human Alport syndrome (71). These renoprotective effects of PDGF-CC were accompanied by a prominent improvement of hypertension in these mice. Additional experiments further supported this finding, suggesting a novel role of PDGF-CC in the regulation of blood pressure by directly acting on arterial vessels and the angiotensin system (71). The second PDGF receptor, PDGR-β, is another well-known mediator of fibrosis. The upregulation of PDGFRβ, a marker of mesenchymal cells, is a well-recognized characteristic of renal fibrosis. It has recently been shown that both in animal models and in patients with renal fibrosis PDGFRβ is not only upregulated but also significantly strongly activated, i.e., phosphorylated (72). To study the consequences of PDGFRβ activation, a novel transgenic model was developed, in which a constitutively active PDGFRβ mutant was specifically expressed in renal mesenchymal cells using the FoxD1 promotor (72). These mice developed renal fibrosis in both glomeruli and the interstitium, which at higher age led to a deterioration of renal function. This is the first model of pure fibrosis, i.e., without underlying epithelial or endothelial injury, inflammation or hypertension. It proved for the first time that fibrosis *per se* is harmful and leads to organ damage and allows truly specific testing of anti-fibrotic drugs. Testing of such drugs revealed that interstitial fibrosis might be reversible, while glomerulosclerosis seems irreversible. Interstitial renal cells are responsible for the production of erythropoietin, an essential hormone regulating erythropoiesis, and in CKD erythropoietin production is reduced resulting in renal anemia. Similar to CKD patients, the transgenic mice with PDGFRβ activation had reduced erythropoietin production and developed progressive anemia. Also, the gene signature induced by PDGFRβ in transgenic mice mirrored changes observed in patients with CKD and fibrosis, representing another important translational feature of this model.

Macrophage migration inhibitory factor (MIF), is a potent and well-known pro-inflammatory cytokine. In most cases, MIF was shown to drive inflammation and organ injury *via* actions on inflammatory cells in kidneys and other organs (73). The effects of MIF on resident cells in the kidneys were not well known. In glomeruli, MIF was shown to directly mediate the pathological proliferation of mesangial and parietal epithelial cells, thereby driving mesangioproliferative and extracapillary proliferative (crescentic) glomerulonephritis (74). Similar effects were observed for CD74, one of the MIF receptors, suggesting that the above MIF effects might be mediated *via* CD74. Besides, the effects of MIF were additive to those of PDGF, which is a well-known mitogen for mesangial cells. Surprisingly however, using several ways to manipulate MIF in a comprehensive set of models of interstitial injury and renal fibrosis, MIF was found to significantly limit rather than promote both renal fibrosis and inflammation (75). These protective effects seemed to be mediated locally by renal tubular cells, as confirmed by specific tubular cell deletion experiments. Similar to glomeruli, MIF increased tubular cell proliferation, but in contrast to glomeruli, in the tubulointerstitium and CKD, the induction of tubular cell proliferation is a desired, regenerative process. Additionally, MIF limited tubular cell cycle arrest, another pathological process contributing to renal fibrosis. Given that tubular injury and (programmed) cell death are hallmarks of acute kidney injury (AKI), and tubular cell regeneration is an essential process in healing, the role of MIF was also analyzed in this setting (76). Clinical observational studies showed that in patients after cardiac surgery, who often develop AKI, higher MIF was associated with reduced prevalence and severity of AKI, confirming renoprotective MIF effects. In patients after partial tumor nephrectomy, circulating MIF concentrations were associated with the length of kidney ischemia during the surgery, suggesting that MIF was released during renal ischemia. Murine experiments showed that already a single administration of recombinant MIF limited tubular injury and particularly programmed cell death, thereby improving the course of models of AKI, and these renoprotective effects were confirmed in *in vitro* studies. Taken together, these studies suggested a dual role of MIF, promoting inflammatory and glomerular diseases but limiting chronic and acute kidney diseases. Potential future treatment approaches will therefore attempt to find ways of how to target MIF to specific cells and disease entities.

Albeit quite common, the mechanisms of crystal-induced nephropathies only start to be revealed. Crystals forming in renal tubular lumens undergo various fates, namely clearance by excretion in the urine, uptake by tubular cells or they may obstruct the whole tubular lumen, the later leading to nephron loss. In addition, an interesting active cellular mechanism of crystal clearance, termed extratubulation of crystals, was described, in which tubular cells overgrow larger crystals and “push” them into the interstitium, thereby reconstituting the tubular lumen (77). However, in the interstitium, the crystals induce a granulomatous (foreign-body) inflammatory reaction leading to degradation of the crystals. Concerning molecular mechanisms, in an animal model of 2,8-dihydroxyadenine nephropathy, only Tnfr1 deficiency ameliorated disease course and renal fibrosis, whereas deficiency in Tnfr2, CD44 or fetuin-A, all of which are involved in the pathogenesis of calcium oxalate nephropathy, had no benefit (77). These data suggested that while some pathogenic mechanisms might be common for all types of crystals, e.g., inflammasome activation, others might be more crystal-type specific. Another important type of crystal-induced kidney injury, localized in the arterial vessels and not tubules, is cholesterol crystal embolism (CCE), a potential adverse event of endovascular interventions (78). In a novel murine model of CCE, a comprehensive assessment revealed crystal clots, and particularly fibrin, extracellular DNA and platelets as major determinants of kidney damage. The model also enabled the analysis of the most promising therapeutic window before or during the endovascular intervention in cardiology or cardiovascular surgery (78). These data therefore considerably extend our understanding of mechanisms of organ injury in important and common crystal-induced diseases.

One aspect hindering the translation of novel anti-fibrotic approaches, such as those described above, to the clinic is the lack of diagnostic approaches that would enable a specific, quantitative and non-invasive measurement of renal fibrosis (79). First preclinical experiments showed that molecular imaging of the extracellular matrix components elastin and collagen might be such novel diagnostic approaches (57, 58).

Renal biopsies remain an essential tool for diagnostics of kidney diseases, and currently remain the only way to specifically assess renal fibrosis. However, the analyses of renal biopsies, particularly any quantitative or semiquantitative measurements and scorings, rely on the subjective judgment of a nephropathologist. Digitalization of pathology enables to improve objectivity, precision, reproducibility and quantitative aspect of pathology toward a computational digital pathology as a tool of precision medicine (80). Especially the use of artificial intelligence and more specifically deep learning opens many new possibilities of augmented computational pathology diagnostics. The most recent developments were in the field of oncological pathology, given the available and well-annotated datasets. In colorectal cancer, deep learning was shown to be able to predict molecular alterations, i.e., microsatellite instability, from a single HE stained histological section with high accuracy (81, 82). Such deep learning-based *in vitro* diagnostics could significantly accelerate molecular tumor diagnostics for negligible cost compared to the current approaches of molecular pathology. Also in nephropathology, new data show the potential of deep learning augmented assessment of kidney histology (80, 83). Several methods for segmentation of kidney histopathology were described that can deal with small datasets, detection of sparse objects or stain-independent supervised and unsupervised segmentation (84–86). Deep learning approaches to segment major diagnostically relevant kidney compartments showed high accuracy, broad applicability and the potential to derive large-scale, precise quantitative data in high-throughput from kidney histology, opening new possibilities of data mining of morphological data (87).

### Imaging Kidney Fibrosis

Non-invasive imaging is extensively employed for disease diagnosis, staging and treatment monitoring. Unfortunately, for kidney fibrosis, no specific imaging probes and protocols are available in the clinic (79). Consequently, needle-based biopsies have remained to be the gold standard method for disease diagnosis. Biopsies have multiple drawbacks: they are invasive, they suffer from sampling variability, they provide limited spatial information, and they cannot be regularly repeated. The latter makes it difficult to employ them for longitudinal assessment of therapy responses and thus for drug development. There consequently is a clear need to develop materials and methods that allow for non-invasive, disease-specific and readily repeatable assessment of kidney fibrosis stage, progression and treatment response.

**Figure 7:**
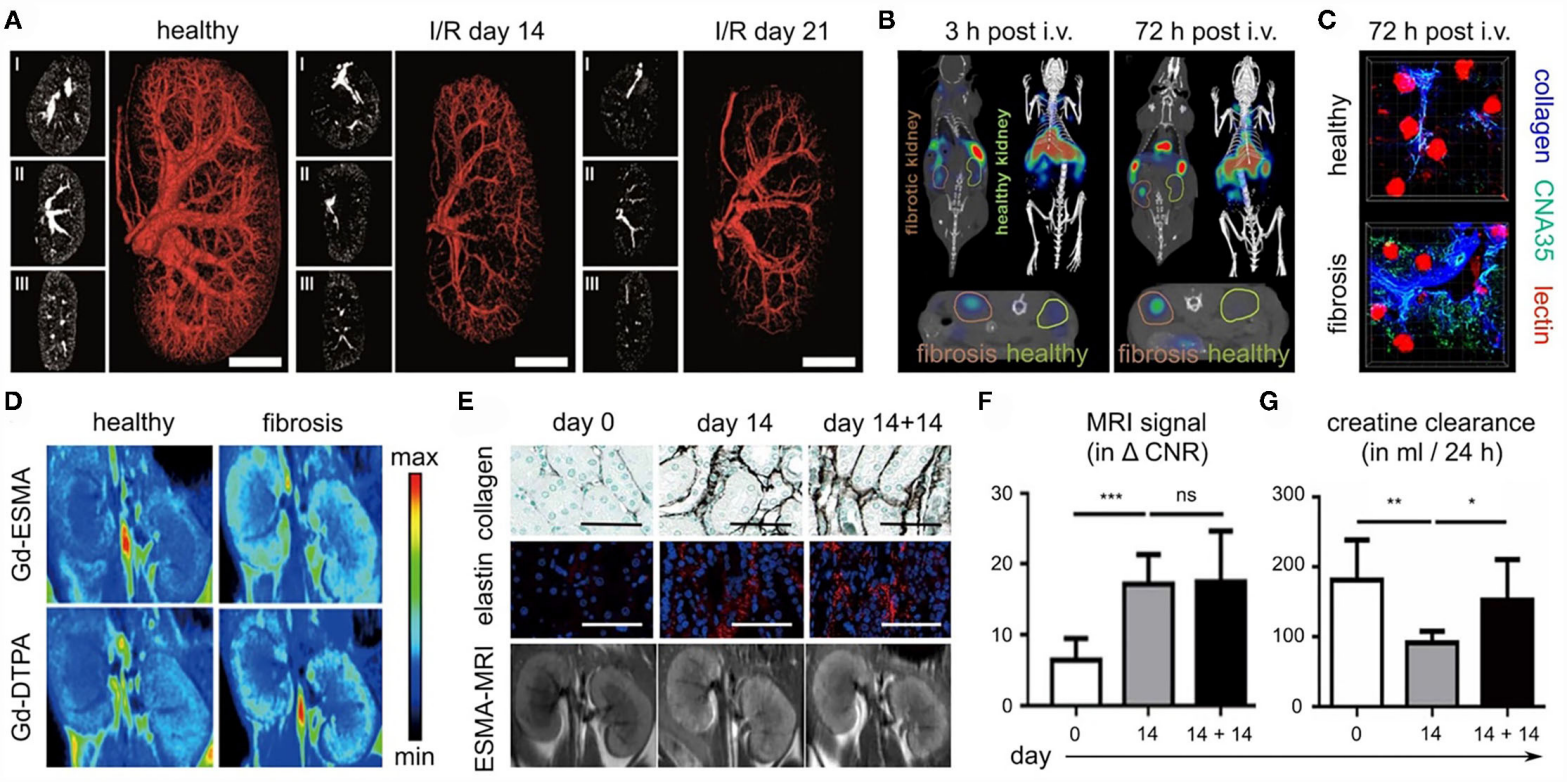
Imaging kidney fibrosis. **(A)** 2D and 3D images reconstructed from contrast-enhanced micro-CT imaging show progressive vessel rarefaction in mice with I/R-induced kidney fibrosis. Scale bar: 200 μm. **(B)** Molecular imaging of collagen deposition in the kidneys of mice with I/R-induced fibrosis, employing CNA35 and CT-FMT. **(C)** Validation of CNA35 binding to perivascular collagen fibers in mice with I/R-induced kidney fibrosis. Perfused blood vessels are stained with lectin. **(D)** ESMA-enhanced molecular MR imaging of elastin deposition in the kidneys of mice with adenine diet-induced fibrosis. **(E–G)** In the adenine reversal model (14 days on adenine diet, then 14 days recovery), ESMA-based molecular MRI can detect residual fibrosis that is not detected by routine kidney function assessment, i.e. analysis of creatinine clearance. Scale bar in **(E)**: 50 μm. Images reproduced, with permission, from (57–59). For assessing statistical significance, as shown in **(F)** and **(G)**, one-way analysis of variance (ANOVA) followed by Bonferroni correction was used. Statistical significance was defined as *p < 0.05; **p < 0.01; ***p < 0.001.

As part of the SFB/TRR57, several strategies for anatomical, functional and molecular imaging of kidney fibrosis have been explored. From an angiographic perspective, *in vivo* and *ex vivo* micro-CT for anatomical and functional blood vessel imaging was developed (59). In three different renal fibrosis mouse models, i.e., I/R, UUO, and Alport, contrast-enhanced *in vivo* CT imaging revealed a progressive reduction of the renal relative blood volume (Figure 7A). This reduction ranged from 20% in early-stage disease to 60% in late-stage CKD. High-resolution *ex vivo* CT imaging revealed significant kidney fibrosis-induced alterations at the level of preglomerular arteries, as exemplified by a reduced vessel diameter, a reduction in vessel branching and an increase in vessel tortuosity. These findings and technologies might be useful for evaluating the efficacy of therapies targeting the vasculature in CKD, particularly at the preclinical level.

Renal fibrosis is characterized by the excessive deposition of extracellular matrix proteins, such as collagen elastin. Several of our efforts have focused on the development and testing of probes for specific molecular imaging of renal fibrosis. In a collagen-centered approach (58), multimodal and multiscale optical imaging was employed (88, 89), using a fluorophore-labeled polypeptide contrast agent CNA35 (for a 35 kDa collagen-binding adhesion protein). Using CT-FMT imaging, upon intravenous injection, the accumulation of CNA35 in I/R-induced fibrotic kidneys was found to be significantly higher than in contralateral healthy kidneys (Figure 7B). *Ex vivo* two-photon microscopy confirmed the strong and specific localization of CNA35 in fibrotic kidneys as compared to healthy kidneys, hinting toward overlap with perivascular collagen fibers (Figure 7C). When employing a non-specific scrambled version of CNA35, such differences were not observed, confirming the specificity of this approach. When incubating tissue specimens from patients with renal fibrosis with fluorophore-labeled CNA35, it was found that collagen-binding polypeptide co-localized with the fibrosis-associated collagen subtypes I and III, but not with collagen IV, which is located in the basement membrane. Taken together, the results showed that non-invasive and quantitative collagen imaging in mice is feasible (58). Also, the imaging helped to highlight that the process of perivascular fibrosis, in addition to the more commonly known interstitial fibrosis, is a key feature of kidney fibrosis.

As a second strategy for molecular imaging of kidney fibrosis, the extracellular matrix protein elastin was addressed (57). Among the main rationales for this is the notion that elastin is present at much lower levels in healthy kidneys than collagen, which could help to create a sharper diagnostic differentiation between diseased and healthy kidneys. In addition, elastin may have a larger dynamic range in terms of assessing disease progression and therapy responses. Elastin expression was hardly detectable in healthy kidneys in mice, rats and humans. Conversely, it was found to be highly upregulated in cortical, medullar and perivascular regions in progressive CKD. These findings were validated in 10 CKD animal models and in 12 major human renal pathologies. To visualize elastin expression, the elastin-specific MRI contrast agent ESMA was employed, which had been employed before for imaging atherosclerosis (90) and liver fibrosis (91). In the case of kidney fibrosis, ESMA-enhanced T_1_-weighted MRI and relaxometry analysis revealed significant differences in fibrotic vs. healthy kidneys, which were stronger than the differences observed for the untargeted MRI contrast agent Gd-DTPA (Figure 7D). In multiple mouse models, evidence was obtained showing that elastin imaging can be employed for non-invasive disease staging. In addition, ESMA-enhanced molecular MRI allowed for repetitive and reproducible assessment of renal fibrosis therapy, as verified upon treatment with the inflammasome inhibitor CRID3 and with the kinase inhibitor imatinib. Also, elastin imaging could detect residual features of fibrosis after kidney injury removal and kidney function normalization (57). For this, a reversible renal injury model was employed, which relies on a first phase of 14 days of adenine diet, resulting in fibrosis development, and then a second phase of 14 days in which the adenine diet is removed, and in which the kidney can gradually recover from the injury. After the 14-day recovery phase, the kidney was histopathologically still found to be damaged, as exemplified by the high levels of collagen and elastin deposition that remained to be present in the kidney (Figure 7E). These residual features of fibrosis could be visualized and quantified by the elastin-specific molecular MRI (Figures 7E,F). In contrast, the kidney function of the mice appeared to have normalized, based on multiple clinically used kidney function readouts, including creatinine clearance (Figure 7G). This showed the potential value of molecular imaging for assessment of renal fibrosis stage and antifibrotic therapy response without the need of having to perform biopsies.

Taken together, significant progress has been made in the last couple of years with regard to the development of probes and protocols for non-invasively visualizing and quantifying kidney fibrosis (79, 92). The resulting functional and (particularly) molecular imaging biomarkers are useful for disease diagnosis, staging and treatment monitoring. Because they can serve as surrogate endpoints in clinical trials in which novel antifibrotic drugs (and other anti-CKD agents) are being tested, imaging biomarkers are considered to be valuable for facilitating translational research.

## Perspectives

Renal fibrosis, the common endpoint of almost all progressive kidney diseases, is not a simple, uniform scarring, but a dynamic process involving many, if not all, renal and infiltrating cell types. Concerning targeted therapies to modulate the processes involved in the development of renal fibrosis, which go beyond the regulation of the patients' blood pressure and broad immunosuppressive therapies, many aspects have not yet been adequately clarified. Some fibrosis-associated factors and signaling pathways, however, which were previously very well characterized in numerous preclinical studies, have failed as targets in subsequent clinical trials. Causal therapeutic measures for patients to specifically reduce established kidney fibrosis or to stop fibrosis progression are therefore still not available. The most prominent example of a fibrosis-related target that was successfully verified in many baseline studies but later used with little or no therapeutic effects in clinical trials with patients with diabetic nephropathy or FSGS is transforming growth factor (TGF)-β (93, 94). The reasons are manifold and may also be due to the design of the studies [reviewed in Isaka (95)]. This and other examples made the pharmaceutical industry reluctant to invest in tedious and expensive trials to cure kidney patients from fibrosis. Next to the lack of good fibrosis models exactly mimicking the human situation, two of the major obstacles to more clinical trials in nephrology are long study times and the lack of appropriate, well-defined endpoints. One of the projects addressed by the SFB/TRR57 “Organ fibrosis–From Mechanisms of Injury to Modulation of Disease” introduced the molecular imaging for noninvasive visualization and quantification of matrix deposition in renal fibrosis. The transfer of this method to the clinic could provide novel endpoints for clinical trials, that could subsequently be realized in shorter periods of time, and thereby, in addition to an improved management of patients with CKD, become significantly more attractive for the pharmaceutical industry. Other projects addressed by the SFB/TRR57 made significant contributions to the understanding of new underlying mechanisms of the initiation, progression and modulation of renal fibrosis. New techniques, such as scRNA-seq analyzes, identified new fibrosis-relevant subsets of involved myofibroblasts and matrix-producing tubular cells. For some of these subsets it is not yet clear if they promote fibrosis or if they are already part of a regenerative program that could serve as a new and more specific therapeutic approach in the future. Notably, analogies of relevant signaling pathways and/or pathomechanisms of organ fibrosis were uncovered between the kidney and the liver (e.g., the role of glomerular parietal cells and hepatic stellate cells). There are good reasons to believe that the identification of new treatment targets to limit renal fibrosis progression and especially the identification of new, reliable fibrosis biomarkers and new methods for successful non-invasive imaging will contribute to bridging the gap between basic science and clinical practice, thereby making the initiation of more and new clinical nephrological studies more attractive and practicable.

## Author Contributions

All authors listed have made a substantial, direct, and intellectual contribution to the work and approved it for publication.

## Funding

The SFB/TRR57 was funded by the German Research Foundation during the years 2009–2021.

## Conflict of Interest

The authors declare that the research was conducted in the absence of any commercial or financial relationships that could be construed as a potential conflict of interest.

## Publisher's Note

All claims expressed in this article are solely those of the authors and do not necessarily represent those of their affiliated organizations, or those of the publisher, the editors and the reviewers. Any product that may be evaluated in this article, or claim that may be made by its manufacturer, is not guaranteed or endorsed by the publisher.
